# Hydroxychloroquine overcomes cabergoline resistance in a patient with Lactotroph Pituitary neuroendocrine tumor: a case report

**DOI:** 10.3389/fendo.2022.955100

**Published:** 2022-08-02

**Authors:** Shaojian Lin, Changxi Han, Xiaohui Lou, Zhe Bao Wu

**Affiliations:** ^1^ Department of Neurosurgery, Center of Pituitary Tumor, Ruijin Hospital, Shanghai Jiao Tong University School of Medicine, Shanghai, China; ^2^ Department of Neurosurgery, Ruian People’s Hospital, The Third Affiliated Hospital of Wenzhou Medical University, Ruian, China

**Keywords:** case report, cabergoline, hydroxychloroquine, novel therapy, resistant lactotroph pituitary neuroendocrine tumors

## Abstract

**Objective:**

A 22-year-old man complaining erectile dysfunction underwent transsphenoidal surgery for a 2.7 cm sellar mass with total resection and was confirmed at pathology to have a lactotroph pituitary neuroendocrine tumor (PiNET). Postoperatively, the patient’s PRL remained at high level and therefore accepted high-dose dopamine receptor agonist (DA) therapy. After over 3 months of bromocriptine (BRC) (15mg/day) and over 3 years of cabergoline (CAB) (3mg/week) therapy, the patient’s prolactin (PRL) never achieved long-term normalization. He was diagnosed with DA-resistant lactotroph PitNET.

**Method:**

In this study, the patient was given hydroxychloroquine (HCQ) (200 mg/d) and CAB (3 mg/w) in combination for four months. His PRL level was tested by blood test every month.

**Results:**

Taking the combination therapy of HCQ and CAB, the patient’s uncontrolled PRL level was normalized within one month and was maintained at the normal level thereafter. Pituitary magnetic resonance imaging (MRI) images with enhancement showed no recurrence. The patient also regained normal sexual function.

**Discussion:**

This is the first report on the combination of HCQ with CAB for the effective treatment of DA-resistant lactotroph pituitary neuroendocrine tumor in a patient, which might provide a novel treatment strategy for clinical management.

## Introduction

Pituitary neuroendocrine tumors (PitNETs) represent 10–25% of intracranial neoplasms. They can be classified into clinically non-functioning PitNETs and hormone- secreting PitNETs. Lactotroph PiNETs account for 53% of PitNETs (1). Currently, dopamine receptor agonist (DA) including bromocriptine (BRC) and cabergoline (CAB) are the first-line medical treatment to lactotroph PiNET. Our previous review summarizing publications from 2000 to 2018 showed that CAB normalized prolactin (PRL) levels in 79.7% of patients and reduced tumour volume in 73.9% of patients (2). However, the remaining patients showed partial or complete DA resistance. DA resistance is defined by inability to achieve PRL normalization or 30% shrinkage in tumor diameter imageologically after three to six consecutive months administration of over 15 mg/day of BRC or 2 mg/week of cabergoline, according to a review published in 2019 (3). These patients need more invasive therapy such as transsphenoidal surgery and radiotherapy, which may cause huge influence on their life quality. There is an unmet demand for the effective treatment of patients with DA-resistant lactotroph PiNETs.

Hydroxychloroquine (HCQ)/chloroquine (CQ) is traditionally used for rheumatoid arthritis, systemic lupus erythematosus, and malaria. Severe side effects include nausea and vomiting (10%), dermatology affections (6%), cardiac complications (3%), visual complaints (2%), and others (4). Compared with CQ, HCQ has lower toxicity and causes less side effects (5). Our previous studies have demonstrated that chloroquine (CQ) could increase the sensitivity of PitNETs to CAB by inhibiting autophagy *in vitro* (6). Here, for the first time, we report a patient who was resistant to DA treatment is finally cured by the combination of HCQ and CAB.

## Case presentation

**Figure 1:**
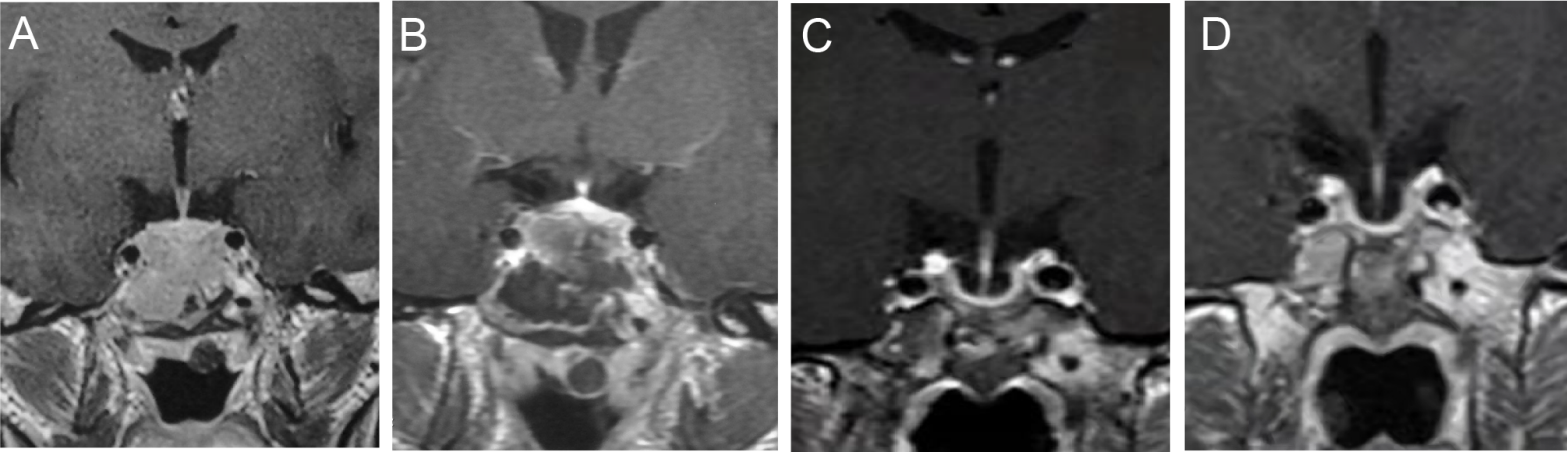
T1WI by contrast enhanced MRI before, after the surgery and in the follow up. MRI before the surgery **(A)**. One week after the surgery **(B)**. Four months before HCQ and CAB combination treatment **(C)**. Three months after HCQ and CAB combination treatment **(D)**.

A 22-year-old man came to the hospital in 2010 with complaints of erectile dysfunction and occasional mild visual dysfunction for two years, with no headache and galactorrhea, and his symptoms had worsened in the last two months. During this period, no clinical treatment had been given to the patient. Physical examination showed no other abnormal signs. Perimetry showed no visual field defect. Hypophysial hormone testing showed a serum PRL level of over 21,186.44 mIU/L (normal range: 55.96-278.36 mIU/L). Contrast enhanced magnetic resonance imaging (MRI) revealed a 2.0×2.7 cm mass in the sella, invading into the sphenoid sinus without surrounding the bilateral internal carotid arteries (**Figure 1A**). The patient chose to have the tumour removed by transsphenoidal surgery. At pathology, PitNETs was confirmed and lactotroph PiNET was diagnosed by immunohistochemistry after surgery (PRL (+), TSH (thyroid stimulating hormone) (-), CK (cytokeratin) (+), ACTH (adrenocorticotropic hormone) (-), LH (luteinizing hormone) (-), CgA (chromogranin A) (-), FSH (follicle-stimulating hormone) (-), GH (growth hormone) (-), Syn (synapsin) (+)).

**Figure 2:**
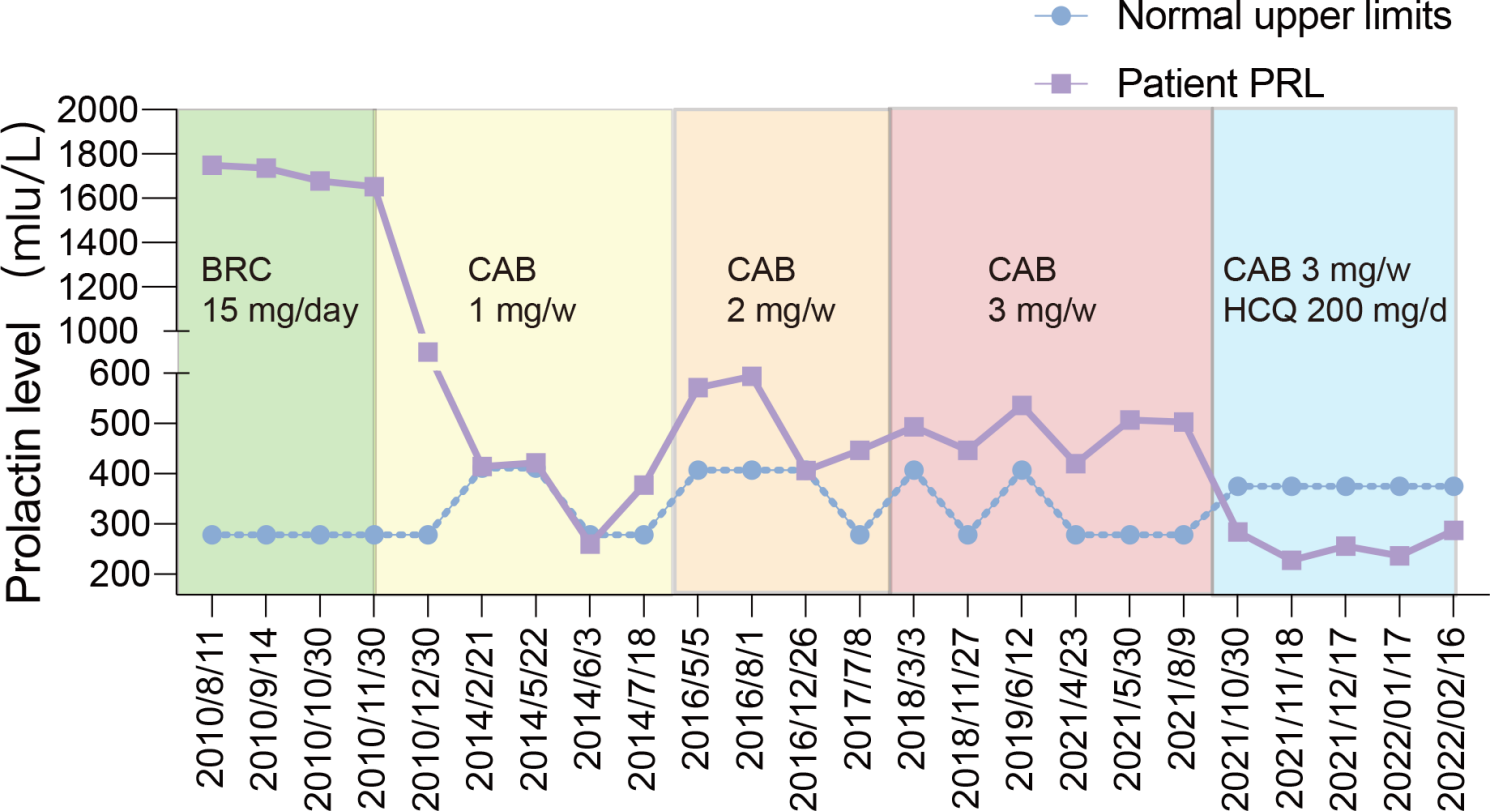
The blue line represented his PRL level in the 12-year following up and the purple line represented the upper limit of PRL normal range according to the hospitals where the blood was tested.

However, the postoperative PRL level of the patient was 1748.59 mIU/L and did not decrease to the normal level despite no residual tumour on MRI (**Figure 1B**). Eventually, the patient accepted dopamine agonist treatment of a gradually increasing dose of BRC from 2.5 mg/day to 15 mg/day. However, even after taking BRC at 15 mg/day for four months, his serum PRL, which was 1737.02 mIU/L, showed no significant decline. Considering the failure to control his serum PRL by BRC, he was recommended to switch to CAB treatment, starting at a dose of 1 mg/week. His blood PRL level decreased to 259.77 mIU/L (within the normal range) after four years of administration, according to the blood test at June 3rd, 2014. One month later, his blood PRL raised again to 377.32 mIU/L (above the upper limit). Then, CAB was increased to 2 mg/week for 3 years and increased to 3 mg/week for 4 years; however, the patient’s PRL level was never normalized (**Figure 2**). MRI showed no residual tumour (**Figure 1C**). Therefore, the patient was diagnosed with DA-resistant lactotroph PiNET.

Our previous study showed that CQ coordinates the effects of CAB and reverses DA resistance *in vitro* and in an animal model (6). With approval from our hospital ethics committee and written informed consent from the patient, we recommended that he receive combination medication of hydroxychloroquine (HCQ) and CAB. He started HCQ at 200 mg/day in combination with the 3 mg/week CAB on September 30, 2021. 200 mg/day is the initial dose of HCQ for other diseases like rheumatoid arthritis and systemic lupus erythematosus suggested by the dispensatory, which is considered safe. After one month of the combination therapy, his PRL level decreased to the normal range (283.80 mIU/L). In the following three months, the PRL level remained within the normal range (**Figure 2**). In addition, the patient regained normal sexual function. To rule out possible side effects of combination therapy, his visceral organs (liver, kidney, heart, retina) were evaluated by blood test, electrocardiogram, ultrasonic cardiogram, and ophthalmoscopy. No damage or side effects were found. Enhanced MRI continued to show no residual tumour (**Figure 1D**).

## Discussion

This is the first report on the combination of HCQ with CAB for the effective treatment of DA-resistant lactotroph PiNET in a patient. His serum PRL decreased to and remained at the normal range with no obvious side effect observed. This might provide a novel treatment strategy for clinical management.

Currently, there are few treatments for drug-resistant prolactin adenomas, including transsphenoidal surgery, radiotherapy, somatostatin analogs and others. Transsphenoidal surgery is the first option for DA-resistant lactotroph PiNETs. After complete removal through transsphenoidal surgery, PRL can decrease to normal levels in 67% of cases, and patients can achieve long-term disease remission (7). However, even when the postoperative MRI shows no residual tumour, the serum PRL levels in some patients cannot achieve long-term normalization. Radiation therapy including stereotactic radiosurgery and fractionated radiation may still be an option for resistant patient who also have failed surgical treatment. However, only 15-50% of the cases might get PRL normalization (8) after a median delay of 3–10 years (3). Meanwhile, radiation therapy could cause hypopituitarism. A study showed that 80% patients developed hypopituitarism 10-15 years after radiation therapy (9, 10). Somatostatin including Octreotide and Lanreotide could provide an option. However, outcomes varied a lot in the current reports, concluded by a review published in 2020 (11). New indicators still need to be discovered to predict the effect of somatostatin analogs on DA resistant lactotroph PiNETs. Recently, metformin was found to inhibit the growth and PRL secretion of lactotroph PiNET cells *in vivo* and *in vitro* (12). However, this was not confirmed in a further study including 10 patients (13).

Recently, HCQ/CQ has been used in the treatment of other solid tumours. A study by Sotelo J et al. found that CQ in combination with radiation therapy and chemotherapy increased the survival rate of glioblastoma patients (14). Another study found that HCQ increased the tumor response to preoperative chemotherapy in pancreatic adenocarcinoma (15). Recently, relevant studies showed that hydroxychloroquine and chloroquine might act through multiple mechanism including inhibition of autophagy and others. Our previous studies have demonstrated that chloroquine (CQ) could increase the sensitivity of PitNETs to CAB by inhibiting autophagy *in vitro* (6). We found that the up-regulation of autophagic protein p26 and LC3-II to the pituitary adenoma cell line, induced by CAB, was prolonged by CQ. This suggested that CQ could cause blockage to the normal autophagic cycles. In addition to autophagy inhibition, King MA et al. found that chloroquine could inhibit the synthesis of cholesterol and lead to cell death (16). Therefore, we registered a clinical trial (NCT03400865) attempting to use CAB with HCQ/CQ to treat the patients with DA-resistant lactotroph PiNET.

Our report suggests that HCQ in combination with CAB provides a novel treatment strategy for DA-resistant lactotroph PiNETs. Stronger evidences will be provided after our clinical trial (NCT03400865) comes into practice in the future.

## Ethics statement

The studies involving human participants were reviewed and approved by Ruijin Hospital Ethic Committee, Shanghai JiaoTong University School of Medicine. The participant provided their written informed consent to participate in this study.

## Author contributions

SL and ZW conceptualized and designed the treatment. XL gathered clinical information of the patient during the follow up. CH and SL drafted the manuscript and the figures. SL, CH and ZW modified the manuscript critically for important content. All authors listed have made a substantial, direct, and intellectual contribution to the work, and approved it for publication.

## Funding

This work was supported by the National Natural Science Foundation of China (grant Nos. 82141114 and 81972339 to ZW and 81701359 to SL).

## Acknowledgments

We gratefully thank Dr. Beverly M K Biller and Xun Zhang of Massachusetts General Hospital for critical reading and editing.

## Conflict of interest

The authors declare that the research was conducted in the absence of any commercial or financial relationships that could be construed as a potential conflict of interest.

The reviewer CL declared a shared affiliation with the authors SL, CH, and ZW to the handling editor at the time of review.

## Publisher’s note

All claims expressed in this article are solely those of the authors and do not necessarily represent those of their affiliated organizations, or those of the publisher, the editors and the reviewers. Any product that may be evaluated in this article, or claim that may be made by its manufacturer, is not guaranteed or endorsed by the publisher.

## References

[1] Daly AF Beckers A . The epidemiology of pituitary adenomas. Endocrinol Metab Clin North Am (2020) 49(3):347–55. doi: 10.1016/j.ecl.2020.04.002 32741475

[2] Lin S Zhang A Zhang X Wu ZB . Treatment of pituitary and other tumours with cabergoline: new mechanisms and potential broader applications. Neuroendocrinology (2020) 110(6):477–88. doi: 10.1159/000504000 31597135

[3] Maiter D . Management of dopamine agonist-resistant prolactinoma. Neuroendocrinology (2019) 109(1):42–50. doi: 10.1159/000495775 30481756

[4] Souza Botelho M Bolfi F Leite R Leite MSF Banzato LR Soares LT . Systematic review and meta-analysis of the safety of chloroquine and hydroxychloroquine from randomized controlled trials on malarial and non-malarial conditions. Syst Rev (2021) 10(1):294. doi: 10.1186/s13643-021-01835-x 34736537 PMC8567984

[5] Ruiz-Irastorza G Ramos-Casals M Brito-Zeron P Khamashta MA . Clinical efficacy and side effects of antimalarials in systemic lupus erythematosus: a systematic review. Ann Rheum Dis (2010) 69(1):20–8. doi: 10.1136/ard.2008.101766 19103632

[6] Lin SJ Wu ZR Cao L Zhang Y Leng ZG Guo YH . Pituitary tumor suppression by combination of cabergoline and chloroquine. J Clin Endocrinol Metab (2017) 102(10):3692–703. doi: 10.1210/jc.2017-00627 28973192

[7] Zamanipoor Najafabadi AH Zandbergen IM de Vries F Broersen LHA van den Akker-van Marle ME Pereira AM . Surgery as a viable alternative first-line treatment for prolactinoma patients. a systematic review and meta-analysis. J Clin Endocrinol Metab (2020) 105(3):e32-e41. doi: 10.1210/clinem/dgz144 PMC7112976 31665485

[8] Loeffler JS Shih HA . Radiation therapy in the management of pituitary adenomas. J Clin Endocrinol Metab (2011) 96(7):1992–2003. doi: 10.1210/jc.2011-0251 21525155

[9] Minniti G Osti M Jaffrain-Rea ML Esposito V Cantore G Maurizi Enrici R . Long-term follow-up results of postoperative radiation therapy for cushing’s disease. J Neurooncol (2007) 84(1):79–84. doi: 10.1007/s11060-007-9344-0 17356896

[10] van den Bergh AC van den Berg G Schoorl MA Sluiter WJ van der Vliet AM Hoving EW . Immediate postoperative radiotherapy in residual nonfunctioning pituitary adenoma: beneficial effect on local control without additional negative impact on pituitary function and life expectancy. Int J Radiat Oncol Biol Phys (2007) 67(3):863–9. doi: 10.1016/j.ijrobp.2006.09.049 17197121

[11] Souteiro P Karavitaki N . Dopamine agonist resistant prolactinomas: any alternative medical treatment? Pituitary (2020) 23(1):27–37. doi: 10.1007/s11102-019-00987-3 31522358 PMC6957547

[12] Gao J Liu Y Han G Deng K Liu X Bao X . Metformin inhibits growth and prolactin secretion of pituitary prolactinoma cells and xenografts. J Cell Mol Med (2018) 22(12):6368–79. doi: 10.1111/jcmm.13963 PMC6237574 30334324

[13] Portari LHC Correa-Silva SR Abucham J . Prolactin response to metformin in cabergoline-resistant prolactinomas: a pilot study. Neuroendocrinology (2022) 112(1):68–73. doi: 10.1159/000514591 33477154

[14] Sotelo J Briceno E Lopez-Gonzalez MA . Adding chloroquine to conventional treatment for glioblastoma multiforme: a randomized, double-blind, placebo-controlled trial. Ann Intern Med (2006) 144(5):337–43. doi: 10.7326/0003-4819-144-5-200603070-00008 16520474

[15] Zeh HJ Bahary N Boone BA Singhi AD Miller-Ocuin JL Normolle DP . A randomized phase II preoperative study of autophagy inhibition with high-dose hydroxychloroquine and Gemcitabine/Nab-paclitaxel in pancreatic cancer patients. Clin Cancer Res (2020) 26(13):3126–34. doi: 10.1158/1078-0432.CCR-19-4042 PMC8086597 32156749

[16] King MA Ganley IG Flemington V . Inhibition of cholesterol metabolism underlies synergy between mTOR pathway inhibition and chloroquine in bladder cancer cells. Oncogene (2016) 35(34):4518–28. doi: 10.1038/onc.2015.511 PMC5000518 26853465

